# A rare case report of Erdheim–Chester disease with pericardial effusion, conduction abnormalities, and atrial infiltration

**DOI:** 10.1093/ehjcr/ytae002

**Published:** 2024-01-04

**Authors:** Charaf Yassine, Giuseppe Colletti, Acasandrei Ciprian, Mairesse George

**Affiliations:** Department of Cardiology, Saint-Joseph Clinic, Route de Lennik 808, Brussels, Belgium; Department of Cardiology, Saint-Joseph Clinic, Route de Lennik 808, Brussels, Belgium; Department of Cardiology, Saint-Joseph Clinic, Route de Lennik 808, Brussels, Belgium; Department of Cardiology, Saint-Joseph Clinic, Route de Lennik 808, Brussels, Belgium

**Keywords:** Erdheim–Chester disease, Pericardial effusion, BRAF(V600E), Dabrafenib, Case report

## Abstract

**Background:**

Erdheim–Chester disease (ECD) is a rare multisystem disorder that primarily affects adults. It is characterized by the excessive production and accumulation of histiocytes, a type of white blood cell, within multiple tissues and organs, including the cardiovascular system. The infiltration of histiocytes can cause a range of cardiovascular symptoms, including pericardial effusion, myocardial infiltration, and heart failure, among others. Despite the potential severity of these cardiovascular manifestations, ECD is often misdiagnosed or underdiagnosed, leading to delays in appropriate treatment and poor outcomes for patients. As such, there is a pressing need for increased awareness and understanding of ECD’s cardiovascular manifestations among clinicians and researchers. This article aims to highlight the importance of considering ECD as a potential underlying cause of cardiovascular complaints and to encourage further investigation into this uncommon but potentially life-threatening condition.

**Case summary:**

A 63-year-old man presented as outpatient complaining of dyspnoea on exertion during the last 3 weeks (New York Heart Association functional class III). He had also experienced a left shoulder and bilateral knee pain over the last 6 months. The patient was found to have a massive pericardial effusion associated with ECD. While pericardial effusions can have various causes, including infection, cancer, and autoimmune disorders, ECD is one potential cause of this condition. Therefore, it is important for clinicians to consider ECD in the differential diagnosis of patients presenting with unexplained pericardial effusions, particularly in the context of other systemic symptoms suggestive of ECD. We discuss about this specific aetiology and the clinical management of this uncommon condition.

**Discussion:**

Erdheim–Chester disease, a non-Langerhans cell histiocytosis, is a rare multisystem disorder. Diagnosis is challenging and should be suspected in the presence of a pericardial effusion with conduction abnormalities with indicators of a multisystem disease.

**Learning points**

- Erdheim–Chester disease (ECD) is a rare disorder that can affect multiple organs, including the heart. It is important to consider ECD in the differential diagnosis of patients presenting with pericardial effusion. Advances in molecular genetic markers and targeted therapies provide hope for improving outcomes for patients with ECD. The article highlights the importance of recognizing cardiac complications in ECD and emphasizes the efficacy of targeted therapies such as dabrafenib in managing this rare disease.
- Patients with pericardial effusion should consider testing for ECD as a differential diagnosis, especially if other symptoms and imaging findings suggest a potential underlying systemic disorder.
- In summary, the article provides a comprehensive overview of ECD and its potential complications, including cardiac involvement.

## Introduction

Erdheim–Chester disease (ECD) is a rare non-Langerhans cell histiocytosis that can affect different organs in the body, leading to a range of symptoms. Recently, a rare case of ECD has been reported where the patient presented with pericardial effusion, conduction abnormalities, and atrial infiltration. Erdheim–Chester disease is a rare form of non-Langerhans cell histiocytosis that can impact multiple systems in the body and is characterized by tissue infiltration of CD68+ CD1a− histiocytes.^1^ This infiltration can lead to various symptoms, depending on the organs and systems affected. Erdheim–Chester disease primarily affects bones, lungs, skin, and retroperitoneum.^1^ However, there have been several reports of cardiac involvement in ECD.^1^ One such case report describes a patient with ECD who presented with pericardial effusion, conduction abnormalities, and atrial infiltration. Cardiac involvement in ECD is often under recognized and carries a poor prognosis, as seen in this case where the patient had right atrial tumour infiltration along with pericardial effusion and conduction abnormalities.^1^ Early diagnosis and management of ECD is crucial to improve patient outcomes, especially in cases with cardiac involvement. The rarity and multisystemic nature of ECD make it a challenging diagnosis, particularly when cardiac involvement is present. Prompt diagnosis and management of this condition are important to ensure the best possible outcomes for patients.^1^ The reported case of ECD with cardiac involvement highlights the need for increased awareness and consideration of cardiovascular symptoms in patients with rare systemic disorders such as ECD.

## Summary figure

**Figure:**
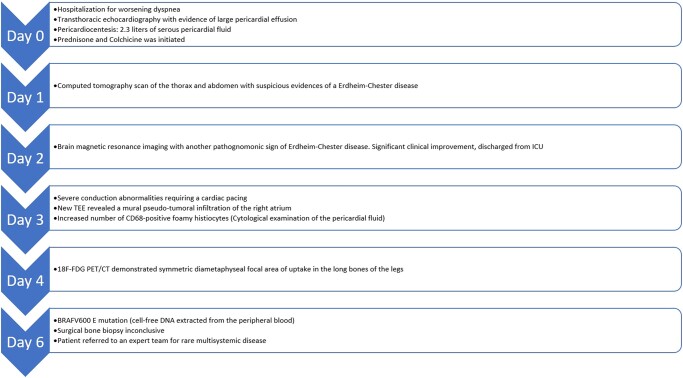


## Case presentation

A 63-year-old male presented at the clinic, reporting shortness of breath during physical exertion for the past three weeks (classified as functional class III according to the New York Heart Association). He also complained of left shoulder and bilateral knee pain persisting for six months. The patient’s medical history included treatment for arterial hypertension with perindopril. No chest pain or viral symptoms were reported before admission.

Upon examination, muffled heart sounds and signs of fluid overload on the right side were observed, including swelling in both lower limbs and mild hepatomegaly. These findings prompted a suspicion of pericardial effusion, later confirmed through transthoracic echocardiography (TEE). Vital signs at the time of the echocardiogram remained stable—blood pressure 150/80 mmHg, heart rate 85 beats/min, and oxygen saturation at 95% while breathing room air.

**Figure 1:**
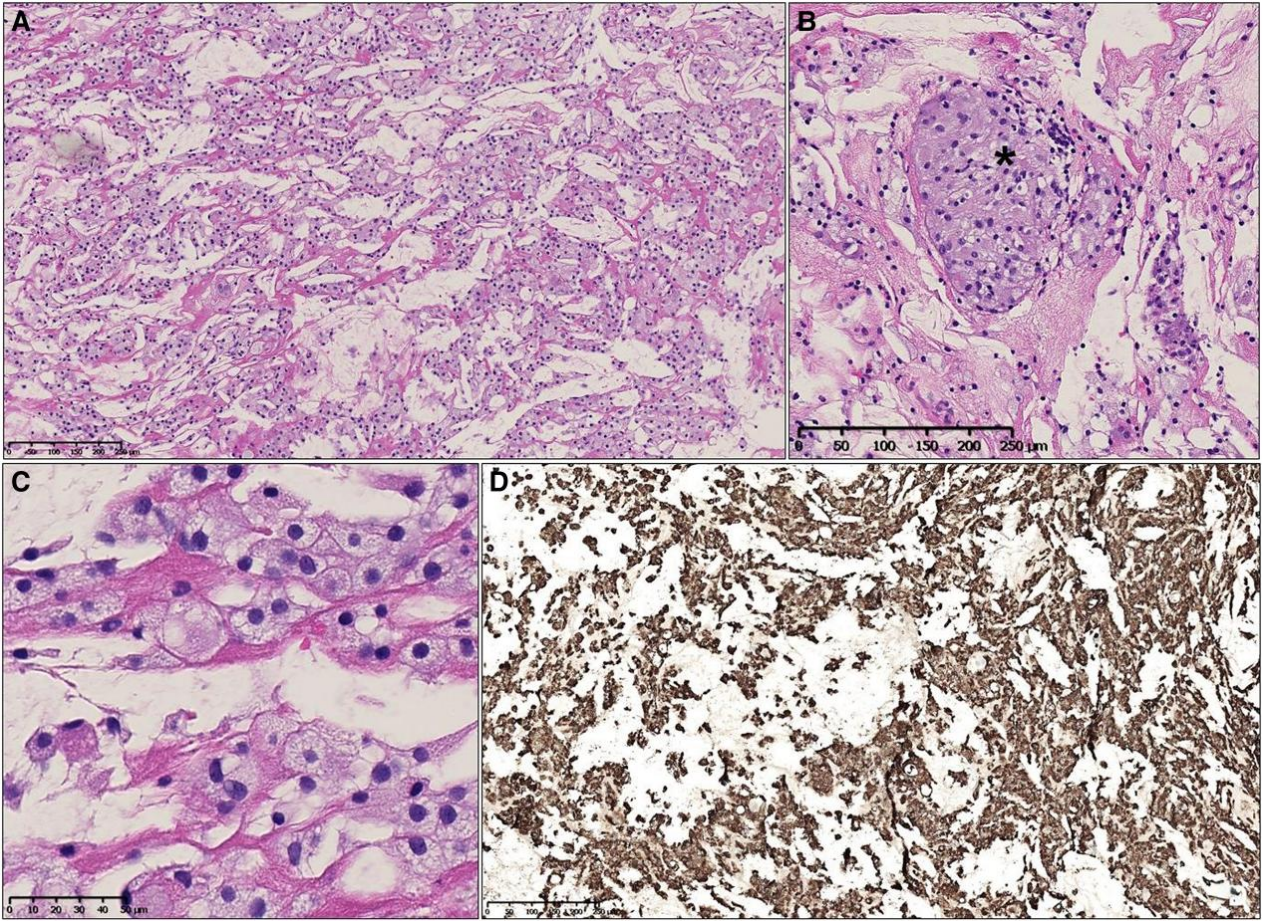
Pericardial fluid cytology. Presence of numerous foamy histiocytes (*A*) forming some granulomatous lesions (*B*, asterisk). The histiocytes do not show nuclear grooves, pseudo inclusions, or emperipolesis (*C*). CD68 positive immunostaining of histiocytes (Cell Block, haematoxylin–eosin, *A*,*B*: 10×, *C*: 40×; immunohistochemical staining, *D*: 10×).

The TEE revealed a large pericardial effusion measuring 40 mm circumferentially around the heart, resulting in partial collapse of the right atrium during early systole and exaggerated respiratory variations of the mitral and tricuspid Doppler flow (see Supplementary material online, videos*S1A*, *B*, and *C* and *S2*). Prompt pericardiocentesis removed 2.3 L of clear fluid, which tested negative for microbial or cancerous cells. The fluid had a total white cell count of 165/mm³ (79% macrophages, 17% lymphocytes) and red blood cell count of 264/mm³. Glucose levels were 119 mg/dL, and total proteins measured 50.32 g/dL (*Figure 1*).

**Figure 2:**
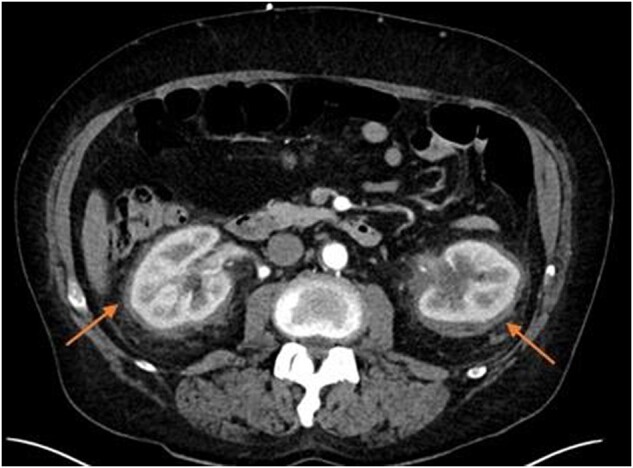
CT of the abdomen showing the typical infiltration of perinephric tissues known as ‘hairy kidney’.

**Figure 3:**
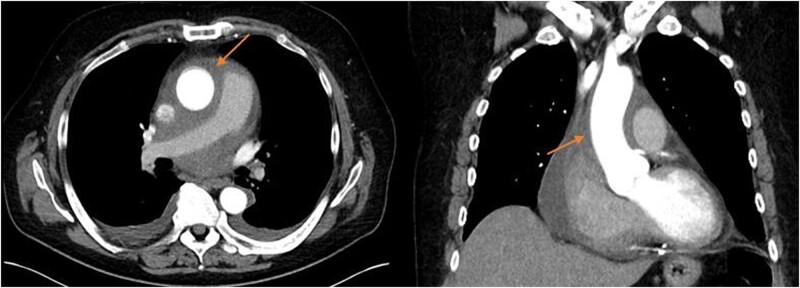
CT of the thorax showing the typical circumferential soft tissue sheathing of the thoracic aorta known as ‘coated aorta’.

Further analysis indicated the presence of foamy histiocytes staining positive with CD68, suggesting ECD. A CT scan confirmed additional signs of ECD, including retroperitoneal fibrosis (hairy kidney) (*Figure 2*) and periadventitial thickening of the thoracic aorta (coated aorta) (*Figure 3*). A brain MRI was performed to assess potential involvement of the central nervous system (CNS) and detect bilateral enhancing lesions orbitally, confirming ECD.

The patient’s condition significantly improved with treatment involving prednisone 64 mg and colchicine 1 mg daily. Prednisone was administered for its anti-inflammatory properties, while colchicine served as an adjunct for inflammation in ECD. Despite improvement, the patient developed severe sinus bradycardia at 30 beats/min, occasionally accompanied by junctional escape rhythm or paroxysmal atrial flutter with slow ventricular conduction. Subsequently, a double-chamber cardiac pacing DDD mode was implanted.

**Figure 4:**
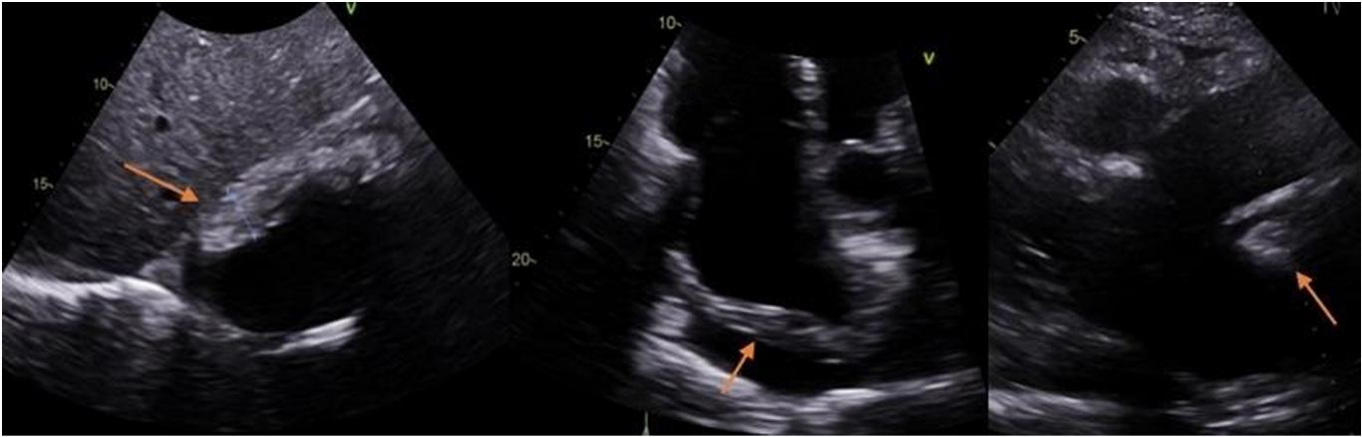
Posterior right atrial pseudotumor (subxiphoid four-chamber, apical four-chamber, and left parasternal long axis views, respectively).

**Figure 5:**
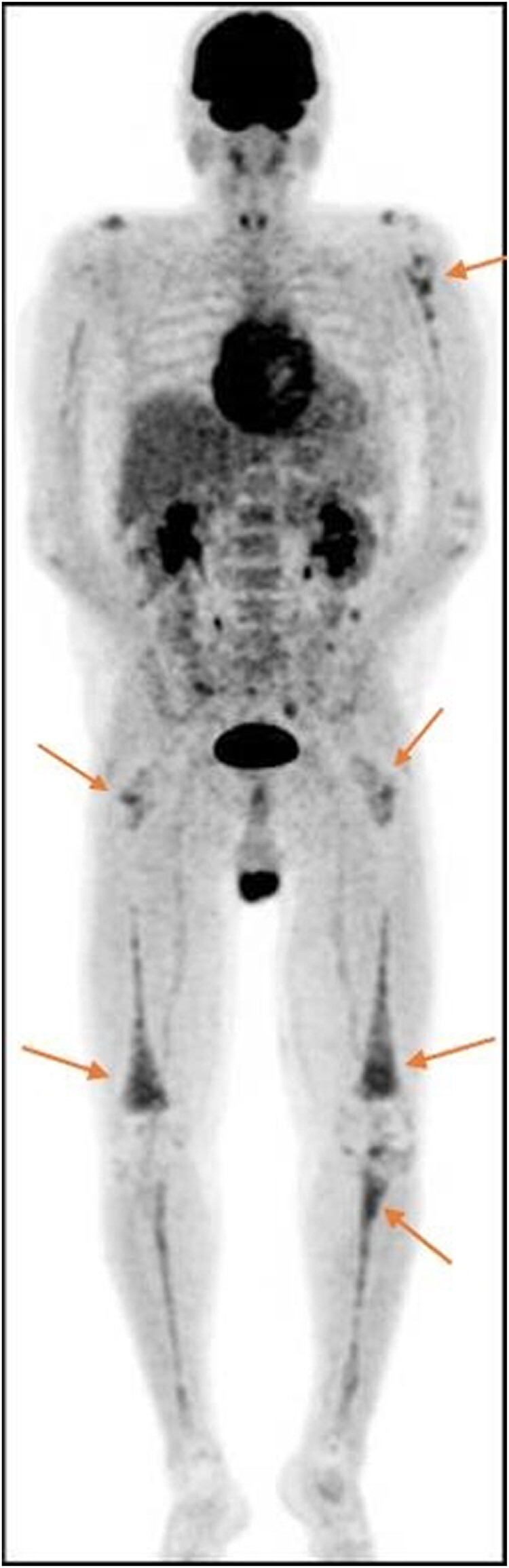
18F-FDG PET/CT demonstrates predominantly epiphyseal 18F-FDG–avid disease.

A follow-up transthoracic echocardiogram revealed evidence of mural pseudo-tumoral infiltration in the right atrium (see *Figure 4* and Supplementary material online, video*S2*). Genetic testing revealed a BRAF gene mutation, specifically BRAFV600E, possibly linked to the presentation. F-18 fluorodeoxyglucose PET/CT scans showed focal areas of uptake on both legs’ long bones symmetrically (refer to *Figure 5*). A surgical procedure to collect bone tissue for analysis did not yield additional diagnostic information.

Following this biopsy, treatment with dabrafenib at a dosage of 150 mg twice daily commenced. This allowed for a gradual discontinuation of corticosteroids over one month after initiating therapy. The patient has remained in good health without experiencing adverse effects, as confirmed during follow-up assessments spanning over 23 months since receiving treatment.

## Discussion

We present a case illustrating ECD, showcasing extensive involvement, particularly affecting the cardiac and vascular systems. The patient exhibited a sizable pericardial effusion, severe conduction abnormalities in the right atria, and involvement of the thoracic aorta. Erdheim–Chester disease is an infrequent non-Langerhans cell histiocytosis, primarily manifesting in the extremities bones, retroperitoneum, CNS, and cardiovascular system. It tends to affect males more frequently and is commonly diagnosed around the sixth decade of life.^2^ Although the aetiology remains a subject of debate, the presence of the BRAFV600E mutation in ∼50% of cases suggests a clonal origin.^3^ Pericardial effusion occurs up to 30% of patients and can result in cardiac tamponade.^4^ Mural pseudo-tumoral infiltration in the right atrium results in conduction abnormalities in up to 30% of cases.^4^ Notably, valvular and coronary artery involvement is rare.^4^ While radiographic features contribute to diagnosis, histological examination is imperative.^5^ Recognizing cardiac complications is crucial, as they are indicative of a poor prognosis.^1^ Cardiac involvement is observed in about 50% of ECD cases, and right atrial tumour is one characteristic finding.^1^ In this case, the patient presented with a right atrial tumour that resulted in severe pericardial effusion and cardiac tamponade requiring emergency intervention. The pathophysiology of pericardial effusion in ECD involves histiocyte infiltration, triggering inflammation and fluid accumulation.^1^ Concurrently, conduction abnormalities and arrhythmias may arise.^1^ Clinicians should consider ECD in patients with unexplained pericardial effusion, particularly those with tamponade, conduction abnormalities, or arrhythmias. Although ECG findings have not been extensively studied, reported abnormalities include arrhythmias and conduction blocks. Prognosis for ventricular function is often poor in cases with cardiac involvement, with possible recurrence of effusion and electrical abnormalities.^1^ Immunochemistry aids in distinguishing ECD from Langerhans cell histiocytosis.^5^ Corticosteroids, though effective in reducing oedema, are not standalone treatments. Recent molecular findings have paved the way for targeted therapies, with BRAF inhibitors like vemurafenib and dabrafenib demonstrating significant efficacy.^6,7^ In our case, dabrafenib was chosen due to the unavailability of vemurafenib in Belgium, leading to enhanced overall survival without notable harm in alignment with the LOVE trial.^8^ While a definitive cure for ECD remains elusive, prior studies of interferon therapy have indicated a five-year survival rate of around 70% among treated patients.^9^ The long-term benefits of targeted drugs in managing ECD complications warrant ongoing research and exploration.

In conclusion, ECD, a non-Langerhans cell histiocytosis, is a rare multisystem disorder. It is characterized by excessive production and accumulation of histiocytes in multiples tissues and organs. Diagnosis is challenging and should be suspected in the presence of a pericardial effusion with conduction abnormalities with indicators of a multisystem disease.

## Supplementary Material

ytae002_Supplementary_Data

## Data Availability

The data underlying this article will be shared on reasonable request to the corresponding author.

## Lead author biography

Dr Charaf Yassine graduated from the Free University of Brussels, Belgium. He currently works as a clinical fellow in the Cardiology Department at Erasmus Hospital, Brussels, Belgium.
